# Characterization of the complete chloroplast genome of *Hordeum vulgare* L. var. *trifurcatum* with phylogenetic analysis

**DOI:** 10.1080/23802359.2021.1935343

**Published:** 2021-06-03

**Authors:** Yuanhang Ren, Hu Xia, Lidan Lu, Gang Zhao

**Affiliations:** Key Laboratory of Coarse Cereal Processing, Ministry of Agriculture and Rural Affairs, Sichuan Engineering and Technology Research Center of Coarse Cereal Industralization, School of Food and Biological Engineering, Chengdu University, Chengdu, P. R. China

**Keywords:** *Tibetan hulless* barley, *Hordeum*, chloroplast genome, phylogenetic analysis, molecular marker

## Abstract

In the present study, the complete chloroplast genome of *Hordeum vulgare* L. var. *trifurcatum* was sequenced, assembled and compared with closely related species. The chloroplast genome of *Hordeum vulgare* L. var. *trifurcatum* was composed of 84 protein-coding genes (PCG), 8 ribosomal RNA (rRNA) genes, and 38 transfer RNA (tRNA) genes. The *Hordeum vulgare L. var. trifurcatum* chloroplast genome is 136,485 bp in size, with the GC content of 38.32%. Phylogenetic analysis based on the combined chloroplast gene dataset indicated that the *Hordeum vulgare* L. var. *trifurcatum* exhibited a close relationship with *Hordeum vulgare* subsp. *spontaneum* and *Hordeum vulgare* subsp. *vulgare*.

*Hordeum vulgare* L. var. *trifurcatum*, belonging to the Poaceae family (Soreng et al. 2015; Saarela et al. 2018), is one of the staple foods for Tibetans and an important livestock feed in the Tibetan Plateau (Zeng et al. 2015; Huang et al. 2020). The highland barley (*Hordeum vulgare* L. var. *trifurcatum*) shows good environmental tolerance, which can be successfully planted in high altitude, low temperature, and high salinity places (Walia et al. 2006; El-Esawi et al. 2018; Elsawy et al. 2018). In addition, the highland barley also showed good antioxidant activity (Asif et al. 2020). Thus, *Hordeum vulgare* L. var. *trifurcatum* is a promising nutritious food source and traditional Chinese medicine grows widely in plateau, which concerning by more and more researchers, just like the *Fagopyrum tataricum* (L.) Gaertn (Song et al. 2016; Xiang et al. 2016; Xiang, Ma, et al. 2019; Xiang, Song, et al. 2019) and *Stellera chamaejasme* L. (Ren et al. 2019, 2021a, 2021b). The genus *Hordeum* comprises more than 30 species. Some varieties are also found in this genus (Malysheva-Otto et al. 2006; Forsberg et al. 2019; Hagenblad and Morales 2020; Kumar et al. 2020). Organelle genomes have been widely used in study of taxonomy, evolution and genetics (Wang et al. 2016; Yang et al. 2019; Wang et al. 2020; Li, He, et al. 2020). However, no complete chloroplast genome of *Hordeum vulgare* L. var. *trifurcatum* was reported to date, which limits its breeding and application (Su et al. 2020).

The specimen (*Hordeum vulgare* L. var. *trifurcatum*) used for chloroplast genome assembly was collected from Qinghai, China (101.97 E; 53.70 N). A specimen was deposited at Collection Center of Chengdu University (Y. Ren, renyuanhang@cdu.edu.cn) under the voucher number ZQK_R1. The chloroplast genome of *Hordeum vulgare* L. var. *trifurcatum* was sequenced and assembled according to methods previously described (Li, Ren, et al. 2020). First, we extracted the total genomic DNA of *Hordeum vulgare* L. var. *trifurcatum* using a Plant DNA Kit (D3485-00, Omega Bio-Tek, Norcross, GA, USA). Then we purified the genomic DNA using a Gel Extraction Kit (Omega Bio-Tek, Norcross, GA, USA). The purified DNA was stored in Chengdu University (No. DNA_ ZQK_R1). Sequencing libraries of *Hordeum vulgare* L. var. *trifurcatum* was constructed using a NEBNext® Ultra™ II DNA Library Prep Kit (NEB, Beijing, China). We conducted the whole genomic sequencing (WGS) of *Hordeum vulgare* L. var. *trifurcatum* using the Illumina HiSeq 2500 Platform (Illumina, SanDiego, CA). The chloroplast genome of *Hordeum vulgare* L. var. *trifurcatum* was initially assembled using SPAdes v3.11.0 (Bankevich et al. 2012). The chloroplast sequences obtained in the above steps were used as seed sequences to assemble the complete chloroplast genome of *Hordeum vulgare* L. var. *trifurcatum* using NOVOPlasty v4.3.1 with a k-mer size of 35 (Dierckxsens et al. 2017). Approximately 1.10 million reads were assembled into a complete circular chloroplast genome. The average chloroplast sequence coverage was 2,168 ×. The complete chloroplast genome of *Hordeum vulgare* L. var. *trifurcatum* was annotated by GeSeq (Tillich et al. 2017) using the chloroplast genome of *Hordeum vulgare* subsp. *spontaneum* as the reference (Bdolach et al. 2019).

The complete chloroplast genome of *Hordeum vulgare* L. var. *trifurcatum* is 136,485 bp in length, which was larger than *Hordeum vulgare* subsp. *vulgare* (136,462 bp) (Zeng et al. 2017) and smaller than *Hordeum vulgare* subsp. *spontaneum* (136,536 bp) (Bdolach et al. 2019). The GC content of the *Hordeum vulgare* L. var. *trifurcatum* chloroplast genome is 38.32%, which is larger than that of *Hordeum vulgare* subsp. *spontaneum* (38.30%). The base compositions of the *Hordeum vulgare* L. var. *trifurcatum* chloroplast genome were as follows: A (30.93%), T (30.76%), G (19.22%) and C (19.10%). The complete chloroplast genome of *Hordeum vulgare* L. var. *trifurcatum* contains 84 protein-coding genes, 8 ribosomal RNA genes, and 38 transfer RNA (tRNA) genes. The number of protein-coding genes in *Hordeum vulgare* L. var. *trifurcatum* chloroplast genome was more than that in two subspecies (*Hordeum vulgare* L. var. *trifurcatum* and *Hordeum vulgare* subsp. *vulgare*), while the number of tRNA was less than that in the two subspecies. To investigate the phylogenetic status of *Hordeum vulgare* L. var. *trifurcatum*, we constructed a phylogenetic tree for 15 species. The protein-coding region of 13 genes conserved in the 15 species was used to construct combined a chloroplast gene set according to previous methods (Li, Xiang, et al. 2019; Wu et al. 2021). Bayesian (BI) analysis method (Li, Wu, et al. 2021) was used to construct the phylogenetic tree based on combined protein-coding genes of chloroplast genome as described by previous methods (Li, Yang, et al. 2020; Cheng et al. 2021; Li, Li, et al. 2021). MrBayes v3.2.6 (Ronquist et al. 2012) was used to construct the phylogenetic tree using Bayesian inference (BI) method. Two independent runs with four chains (three heated and one cold) each were conducted simultaneously for 2 × 10^6^ generations. Each run was sampled every 100 generations. We assumed that stationarity had been reached when estimated sample size (ESS) was greater than 100, and the potential scale reduction factor (PSRF) approached 1.0. The first 25% samples were discarded as burn-in, and the remaining trees were used to calculate Bayesian posterior probabilities (BPP) in a 50% majority-rule consensus tree (Li, Ren, et al. 2019). According to the phylogenetic tree (Figure 1), the *Hordeum vulgare* L. var. *trifurcatum* exhibited a close relationship with *Hordeum vulgare* subsp. *spontaneum* (Bdolach et al. 2019) and *Hordeum vulgare* subsp. *vulgare* (Zeng et al. 2017).

**Figure 1.**
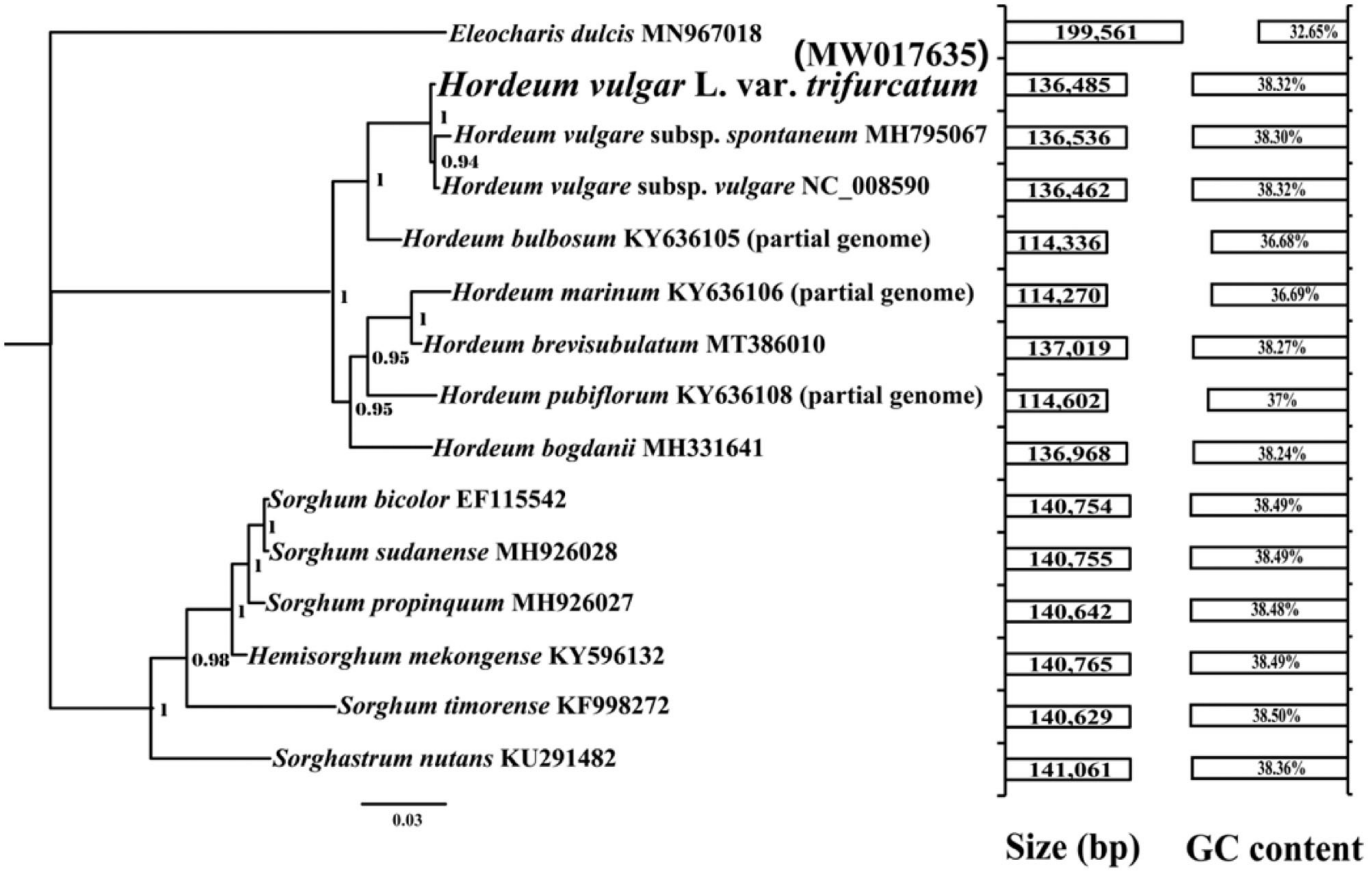
Bayesian phylogenetic analysis and comparative chloroplast genomic analysis of 15 species based on the combined protein-coding gene sets. Support values are Bayesian posterior probabilities (BPP). Accession numbers of chloroplast sequences used in the phylogenetic analysis are listed after the species names.

## Data Availability

The genome sequence data that support the findings of this study are openly available in GenBank of NCBI at (https://www.ncbi.nlm.nih.gov/) under the accession no. MW017635. The associated BioProject, SRA, and Bio-Sample numbers are PRJNA717119, SRR14082750, and SAMN18478699, respectively.

## References

[CIT0001] Asif A, Zeeshan N, Mehmood S. 2020. Antioxidant and antiglycation activities of traditional plants and identification of bioactive compounds from extracts of *Hordeum vulgare* by LC-MS and GC-MS. J Food Biochem. 44(9):e13381. 32696536 10.1111/jfbc.13381

[CIT0002] Bankevich A, Nurk S, Antipov D, Gurevich AA, Dvorkin M, Kulikov AS, Lesin VM, Nikolenko SI, Pham S, Prjibelski AD, Pyshkin AV, et al. 2012. SPAdes: a new genome assembly algorithm and its applications to single-cell sequencing. J Comput Biol. 19(5):455–477. 22506599 10.1089/cmb.2012.0021 PMC3342519

[CIT0003] Bdolach E, Prusty MR, Faigenboim-Doron A, Filichkin T, Helgerson L, Schmid KJ, Greiner S, Fridman E. 2019. Thermal plasticity of the circadian clock is under nuclear and cytoplasmic control in wild barley. Plant Cell Environ. 42(11):3105–3120. 31272129 10.1111/pce.13606

[CIT0004] Cheng J, Luo Q, Ren YH, Luo Z, Liao WL, Wang X, Li Q. 2021. Panorama of intron dynamics and gene rearrangements in the phylum Basidiomycota as revealed by the complete mitochondrial genome of *Turbinellus floccosus*. Appl Microbiol Biotechnol. 105(5):2017–2032. 33555361 10.1007/s00253-021-11153-w

[CIT0005] Dierckxsens N, Mardulyn P, Smits G. 2017. NOVOPlasty: de novo assembly of organelle genomes from whole genome data. Nucleic Acids Res. 45(4):e18. 28204566 10.1093/nar/gkw955 PMC5389512

[CIT0006] El-Esawi MA, Alaraidh IA, Alsahli AA, Ali HM, Alayafi AA, Witczak J, Ahmad M. 2018. Genetic variation and alleviation of salinity stress in barley (*Hordeum vulgare* L.). Molecules. 23(10):2488. 10.3390/molecules23102488 PMC6222302 30274189

[CIT0007] Elsawy HIA, Mekawy AMM, Elhity MA, Abdel-Dayem SM, Abdelaziz MN, Assaha DVM, Ueda A, Saneoka H. 2018. Differential responses of two Egyptian barley (*Hordeum vulgare* L.) cultivars to salt stress. Plant Physiol Biochem. 127:425–435. 29684827 10.1016/j.plaphy.2018.04.012

[CIT0008] Forsberg NEG, Leino MW, Hagenblad J. 2019. Population structure in landrace barley (*Hordeum vulgare* L.) during the late 19th century crop failures in Fennoscandia. Heredity (Edinb). 123(6):733–745. 31616056 10.1038/s41437-019-0277-0 PMC6834677

[CIT0009] Hagenblad J, Morales J. 2020. An evolutionary approach to the history of barley (*Hordeum vulgare*) cultivation in the Canary Islands. Afr Archaeol Rev. 37(4):579–595. 33268912 10.1007/s10437-020-09415-5 PMC7677147

[CIT0010] Huang H, Gao X, Li Y, Tian P, Nima Y, Laba Z, Ci Z, Wei X, Qu J, Guan W, et al. 2020. Content analysis of vitamins, dietary fibers and amino acids in a wide collection of barley (*Hordeum vulgare* L.) from Tibet, China. Bioinformation. 16(4):314–322. 32773991 10.6026/97320630016314 PMC7392089

[CIT0011] Kumar P, Banjarey P, Malik R, Tikle AN, Verma RPS. 2020. Population structure and diversity assessment of barley (*Hordeum vulgare* L.) introduction from ICARDA. J Genet. 99(1):70. 32893841

[CIT0012] Li Q, He X, Ren Y, Xiong C, Jin X, Peng L, Huang W. 2020. Comparative mitogenome analysis reveals mitochondrial genome differentiation in ectomycorrhizal and asymbiotic *Amanita* species. Front Microbiol. 11:1382. 32636830 10.3389/fmicb.2020.01382 PMC7318869

[CIT0013] Li Q, Li LJ, Feng HY, Tu WY, Bao ZJ, Xiong C, Wang X, Huang WL. 2021. Characterization of the complete mitochondrial genome of Basidiomycete yeast *Hannaella oryzae*: intron evolution, gene rearrangement and its phylogeny. Front Microbiol. 12: 646567. 10.3389/fmicb.2021.646567 PMC8193148 34122362

[CIT0014] Li Q, Ren Y, Shi X, Peng L, Zhao J, Song Y, Zhao G. 2019. Comparative mitochondrial genome analysis of two ectomycorrhizal fungi (*Rhizopogon*) reveals dynamic changes of intron and phylogenetic relationships of the subphylum Agaricomycotina. Int J Mol Sci. 20(20):5167. 10.3390/ijms20205167 PMC6829451 31635252

[CIT0015] Li Q, Ren Y, Xiang D, Shi X, Zhao J, Peng L, Zhao G. 2020. Comparative mitogenome analysis of two ectomycorrhizal fungi (*Paxillus*) reveals gene rearrangement, intron dynamics, and phylogeny of basidiomycetes. IMA Fungus. 11(1):12. 32670777 10.1186/s43008-020-00038-8 PMC7333402

[CIT0016] Li Q, Wu P, Li L, Feng H, Tu W, Bao Z, Xiong C, Gui M, Huang W. 2021. The first eleven mitochondrial genomes from the ectomycorrhizal fungal genus (*Boletus*) reveal intron loss and gene rearrangement. Int J Biol Macromol. 172:560–572. 33476615 10.1016/j.ijbiomac.2021.01.087

[CIT0017] Li Q, Xiang D, Wan Y, Wu Q, Wu X, Ma C, Song Y, Zhao G, Huang W. 2019. The complete mitochondrial genomes of five important medicinal *Ganoderma* species: features, evolution, and phylogeny. Int J Biol Macromol. 139:397–408. 31381907 10.1016/j.ijbiomac.2019.08.003

[CIT0018] Li Q, Yang L, Xiang D, Wan Y, Wu Q, Huang W, Zhao G. 2020. The complete mitochondrial genomes of two model ectomycorrhizal fungi (*Laccaria*): features, intron dynamics and phylogenetic implications. Int J Biol Macromol. 145:974–984. 31669472 10.1016/j.ijbiomac.2019.09.188

[CIT0019] Malysheva-Otto LV, Ganal MW, Roder MS. 2006. Analysis of molecular diversity, population structure and linkage disequilibrium in a worldwide survey of cultivated barley germplasm (*Hordeum vulgare* L.). BMC Genet. 7(1):6. 16433922 10.1186/1471-2156-7-6 PMC1408084

[CIT0020] Ren Y, Li Q, Lu L, Jin H, Tao K, Hou T. 2021a. Isochamaejasmin induces toxic effects on *Helicoverpa zea* via DNA damage and mitochondria-associated apoptosis. Pest Manag Sci. 77(1):557–567. 32815281 10.1002/ps.6055

[CIT0021] Ren Y, Li Q, Lu L, Jin H, Tao K, Hou T. 2021b. Toxicity and physiological actions of biflavones on potassium current in insect neuronal cells. Pestic Biochem Physiol. 171:104735. 33357557 10.1016/j.pestbp.2020.104735

[CIT0022] Ren Y, Mu Y, Yue Y, Jin H, Tao K, Hou T. 2019. Neochamaejasmin A extracted from *Stellera chamaejasme* L. induces apoptosis involving mitochondrial dysfunction and oxidative stress in Sf9 cells. Pestic Biochem Physiol. 157:169–177. 31153465 10.1016/j.pestbp.2019.03.025

[CIT0023] Ronquist F, Teslenko M, van der Mark P, Ayres DL, Darling A, Hohna S, Larget B, Liu L, Suchard MA, Huelsenbeck JP. 2012. MrBayes 3.2: efficient Bayesian phylogenetic inference and model choice across a large model space. Syst Biol. 61(3):539–542. 22357727 10.1093/sysbio/sys029 PMC3329765

[CIT0024] Saarela JM, Burke SV, Wysocki WP, Barrett MD, Clark LG, Craine JM, Peterson PM, Soreng RJ, Vorontsova MS, Duvall MR. 2018. A 250 plastome phylogeny of the grass family (Poaceae): topological support under different data partitions. PeerJ. 6 :e4299. 29416954 10.7717/peerj.4299 PMC5798404

[CIT0025] Song C, Xiang DB, Yan L, Song Y, Zhao G, Wang YH, Zhang BL. 2016. Changes in seed growth, levels and distribution of flavonoids during tartary buckwheat seed development. Plant Prod Sci. 19(4):518–527.

[CIT0026] Soreng RJ, Peterson PM, Romaschenko K, Davidse G, Zuloaga FO, Judziewicz EJ, Filgueiras TS, Davis JI, Morrone O. 2015. A worldwide phylogenetic classification of the Poaceae (Gramineae). J Syst Evol. 53(2):117–137.

[CIT0027] Su X, Zhao J, Wang Z. 2020. The complete chloroplast genome of *Hordeum brevisubulatum*. Mitochondrial DNA B Resour. 5(3):2988–2989. 33458027 10.1080/23802359.2020.1797552 PMC7782175

[CIT0028] Tillich M, Lehwark P, Pellizzer T, Ulbricht-Jones ES, Fischer A, Bock R, Greiner S. 2017. GeSeq - versatile and accurate annotation of organelle genomes. Nucleic Acids Res. 45(W1):W6–W11. 28486635 10.1093/nar/gkx391 PMC5570176

[CIT0029] Walia H, Wilson C, Wahid A, Condamine P, Cui X, Close TJ. 2006. Expression analysis of barley (*Hordeum vulgare* L.) during salinity stress. Funct Integr Genomics. 6(2):143–156. 16450154 10.1007/s10142-005-0013-0

[CIT0030] Wang X, Song A, Wang F, Chen M, Li X, Li Q, Liu N. 2020. The 206 kbp mitochondrial genome of *Phanerochaete carnosa* reveals dynamics of introns, accumulation of repeat sequences and plasmid-derived genes. Int J Biol Macromol. 162:209–219. 32562727 10.1016/j.ijbiomac.2020.06.142

[CIT0031] Wang YL, Wei ZX, Xu QJ, Zeng XQ, Yuan HJ, Tang YW, Tashi N. 2016. The complete mitochondrial genome of Tibetan hulless barley. Mitochondrial DNA B Resour. 1(1):430–431. 33490399 10.1080/23802359.2016.1180553 PMC7800387

[CIT0032] Wu P, Bao Z, Tu W, Li L, Xiong C, Jin X, Li P, Gui M, Huang W, Li Q. 2021. The mitogenomes of two saprophytic Boletales species (*Coniophora*) reveals intron dynamics and accumulation of plasmid-derived and non-conserved genes. Comput Struct Biotechnol J. 19:401–414. 33489009 10.1016/j.csbj.2020.12.041 PMC7804350

[CIT0033] Xiang DB, Ma CR, Song Y, Wu Q, Wu XY, Sun YX, Zhao G, Wan Y. 2019. Post-anthesis photosynthetic properties provide insights into yield potential of tartary buckwheat cultivars. Agronomy-Basel. 9(3):149.

[CIT0034] Xiang DB, Song Y, Wu Q, Ma CR, Zhao JL, Wan Y, Zhao G. 2019. Relationship between stem characteristics and lodging resistance of Tartary buckwheat (*Fagopyrum tataricum*). Plant Prod Sci. 22(2):202–210.

[CIT0035] Xiang DB, Zhao G, Wan Y, Tan ML, Song C, Song Y. 2016. Effect of planting density on lodging-related morphology, lodging rate, and yield of tartary buckwheat (*Fagopyrum tataricum*). Plant Prod Sci. 19(4):479–488.

[CIT0036] Yang LX, Li Q, Zhao G. 2019. Characterization of the complete chloroplast genome of *Chenopodium* sp. (Caryophyllales: Chenopodiaceae). Mitochondrial DNA B Resour. 4(2):2574–2575. 33365632 10.1080/23802359.2019.1640089 PMC7706533

[CIT0037] Zeng QX, Yuan JH, Wang LY, Xu JQ, Nyima T. 2017. The complete chloroplast genome of Tibetan hulless barley. Mitochondrial DNA A DNA Mapp Seq Anal. 28(3):324–325. 26713356 10.3109/19401736.2015.1122765

[CIT0038] Zeng X, Long H, Wang Z, Zhao S, Tang Y, Huang Z, Wang Y, Xu Q, Mao L, Deng G, et al. 2015. The draft genome of Tibetan hulless barley reveals adaptive patterns to the high stressful Tibetan Plateau. Proc Natl Acad Sci U S A. 112(4):1095–1100. 25583503 10.1073/pnas.1423628112 PMC4313863

